# IUC24426-83 Predictors of cancer detection on local anesthetic transperineal (LATP) prostate biopsy: a single-center analysis

**DOI:** 10.1093/oncolo/oyaf248.008

**Published:** 2025-08-29

**Authors:** M Ali, M Thomas, M Sanan, J Morton, V Koo

**Affiliations:** Worcestershire Acute Hospitals NHS Trust, United Kingdom; Worcestershire Acute Hospitals NHS Trust, United Kingdom; Worcestershire Acute Hospitals NHS Trust, United Kingdom; Worcestershire Acute Hospitals NHS Trust, United Kingdom; Worcestershire Acute Hospitals NHS Trust, United Kingdom

## Abstract

**Background:**

To evaluate predictors of prostate cancer detection in men undergoing local anesthetic trans-perineal (LATP) prostate biopsy, with a focus on PSA levels and PIRADS scores.

**Methods:**

A retrospective analysis was conducted on 616 patients who underwent LATP biopsy at our single NHS Trust between November 2023 and April 2024. Data collected included PSA, PIRADS score, and histological classification (Cancer, Benign, or Other). Cancer detection rates were analyzed across PSA and PIRADS strata. Management strategies were reviewed for patients with benign histology.

**Results:**

The overall cancer detection rate was 66.8% (412/616). Mean PSA was significantly higher in cancer cases (17.8 ng/mL) compared to benign histology (6.3 ng/mL). PIRADS score correlated strongly with malignancy, with PIRADS 4-5 lesions yielding markedly higher cancer rates than PIRADS 1-3. PIRADS 3 lesions showed mixed outcomes, highlighting diagnostic uncertainty in this group.

Among the 143 patients with benign histology, 46% were discharged from follow-up, while 42% were monitored with repeat PSA testing. Only 3 patients (2%) underwent further investigation, such as repeat biopsy or MRI. No cases of sepsis or major complications were reported.

**Conclusions:**

In this real-world LATP cohort, cancer detection correlated closely with PSA level and PIRADS score. LATP was safe and highly diagnostic, supporting its role as a first-line biopsy strategy. The mixed outcomes of PIRADS 3 lesions and the conservative management of most benign cases highlight the need for refined risk stratification and selective re-biopsy strategies.

